# PlasBin-flow: a flow-based MILP algorithm for plasmid contigs binning

**DOI:** 10.1093/bioinformatics/btad250

**Published:** 2023-06-30

**Authors:** Aniket Mane, Mahsa Faizrahnemoon, Tomáš Vinař, Broňa Brejová, Cedric Chauve

**Affiliations:** Department of Mathematics, Simon Fraser University, Burnaby V5A 1S6, Canada; Department of Mathematics, Simon Fraser University, Burnaby V5A 1S6, Canada; Department of Applied Informatics, Comenius University, Bratislava 84248, Slovakia; Department of Computer Science, Comenius University, Bratislava 84248, Slovakia; Department of Mathematics, Simon Fraser University, Burnaby V5A 1S6, Canada

## Abstract

**Motivation:**

The analysis of bacterial isolates to detect plasmids is important due to their role in the propagation of antimicrobial resistance. In short-read sequence assemblies, both plasmids and bacterial chromosomes are typically split into several contigs of various lengths, making identification of plasmids a challenging problem. In plasmid contig binning, the goal is to distinguish short-read assembly contigs based on their origin into plasmid and chromosomal contigs and subsequently sort plasmid contigs into bins, each bin corresponding to a single plasmid. Previous works on this problem consist of *de novo* approaches and reference-based approaches. *De novo* methods rely on contig features such as length, circularity, read coverage, or GC content. Reference-based approaches compare contigs to databases of known plasmids or plasmid markers from finished bacterial genomes.

**Results:**

Recent developments suggest that leveraging information contained in the assembly graph improves the accuracy of plasmid binning. We present PlasBin-flow, a hybrid method that defines contig bins as subgraphs of the assembly graph. PlasBin-flow identifies such plasmid subgraphs through a mixed integer linear programming model that relies on the concept of network flow to account for sequencing coverage, while also accounting for the presence of plasmid genes and the GC content that often distinguishes plasmids from chromosomes. We demonstrate the performance of PlasBin-flow on a real dataset of bacterial samples.

**Availability and implementation:**

https://github.com/cchauve/PlasBin-flow.

## 1 Introduction

Antimicrobial resistance (AMR) has emerged to become a major threat to public health. AMR is developed in bacteria through the propagation of AMR genes, often facilitated by mobile genetic elements (MGEs), which can be exchanged between bacteria through horizontal gene transfer (HGT; Partridge et al. 2018; De Oliveira et al. 2020). Plasmids are short circular extra-chromosomal MGEs known to be a major vector for the spread of AMR through HGT (Carattoli 2013). Thus, the detection of plasmids in bacterial genomes is an important problem in microbial genomics, with applications in environmental ecology and public health surveillance.

With the advent of DNA sequencing technologies, it is now possible to obtain whole-genome sequencing (WGS) data at a low cost, and the analysis of WGS data for public health surveillance is now common (Gerner-Smidt et al. 2019; Struelens and Sintchenko 2020). Despite the increasing availability of long-read sequencing data (Tedersoo et al. 2021), most surveillance approaches in microbial genomics rely on the analysis of short-read WGS datasets. Such datasets are generally assembled using assemblers such as Unicycler (Wick et al. 2017), SKESA (Souvorov et al. 2018), or SPAdes (Bankevich et al. 2012). It is typically impossible to assemble full sequences of chromosomes and plasmids using short-read WGS data, and consequently, a short-read assembly contains a mixture of contigs coming from the chromosome and individual plasmids present in the sample.

The problem of detecting plasmids from a short-read assembly can be considered at three different levels: classification, binning, and assembly. At the classification level, the aim is to classify the origin of a contig, which is either a plasmid or the chromosome. Current state-of-the-art tools rely on machine-learning approaches and include mlplasmids (Arredondo-Alonso et al. 2018), PlasClass (Pellow et al. 2020), Deeplasmid (Andreopoulos et al. 2022), and RFPlasmid (van der Graaf-van Bloois et al. 2021). However, a bacterial genome can contain several plasmids, and contigs classification does not provide a precise view of the plasmid content of a genome. This is addressed by the plasmid binning problem, where the aim is to group plasmid contigs into bins, such that the contigs in a bin are likely to originate from the same plasmid. Lastly, plasmid assembly aims to order and orient contigs in a plasmid bin into a fully assembled plasmid sequence.

In this work, we focus on the binning problem, motivated by the observation that, for downstream analysis, the most useful information is groups of genes belonging to a given plasmid. While the order and orientation of these genes along the plasmid sequence can yield interesting insights, important tasks such as plasmid typing can be done from the gene content without considering contigs or gene order (Dewar et al. 2021; Luo et al. 2021). Two main methodological avenues have been used in developing plasmid binning methods: reference-based and *de-novo* binning. Reference-based methods, such as MOB-recon (Robertson and Nash 2018), map contigs to a reference database of plasmids or plasmid gene sequences, clustered into families of plasmids. Contigs are then binned together if they match with plasmids belonging to the same reference cluster. The reliance on a reference database can potentially hinder the ability of reference-based methods to identify novel plasmids. *De-novo* binning methods, such as Recycler (Rozov et al. 2017), PlasmidSPAdes (Antipov et al. 2016), and gplas (Arredondo-Alonso et al. 2020), bypass the requirement of reference sequences and rely instead on contig features assumed to be specific to different plasmids present in a bacterial cell. A feature central to many plasmid binning methods is contig read coverage. Different plasmids occur in the genome isolate in varying number (sometimes hundreds) of copies. Thus, the coverage of plasmid contigs is expected to be significantly different between the plasmids and chromosomes, as well as between different plasmids. Another feature used to detect plasmids in a bacterial assembly is the GC content, which is often slightly different in plasmids, especially short ones, as compared to the chromosome (Nishida 2012). Recent plasmid binning methods HyAsP (Müller and Chauve 2019) and PlasBin (Mane et al. 2022) combine ideas from both reference-based and *de-novo* binning.

Several methods do leverage information from the assembly graph, a graph containing contigs as nodes and possible connections between them supported by sequencing data as edges; widely used bacterial genomes assemblers such as SPAdes, Unicycler, and SKESA generate such a graph as a complement to the contigs assembly. The rationale for using the assembly graph is that individual molecules, such as chromosomes or plasmids, ideally correspond to walks in this graph. However, spurious edges and contigs encoding repeats may form densely connected graphs that are difficult to analyse. On the other hand, some edges from such walks can be missing due to low sequencing coverage of the corresponding loci. Nevertheless, our approach assumes that sequencing data is provided at sufficient sequencing depth, resulting in plasmids corresponding to connected subgraphs, often cycles or closed walks, in the assembly graph. This assumption is also central in several other plasmid binning methods. For example, Recycler (Rozov et al. 2017) peels off cycles from the assembly graph assuming uniform coverage of sequenced plasmids; PlasmidSPAdes (Antipov et al. 2016) estimates the chromosomal coverage from the whole assembly, removes contigs with similar coverage as that of the chromosome and then computes putative plasmid bins from the connected components of the remaining graph. Gplas (Arredondo-Alonso et al. 2020) constructs a plasmidome network by defining a set of walks in the assembly graph. Each walk is initialized with a contig i.e. classified as plasmidic using mlplasmids (Arredondo-Alonso et al. 2018). It uses a heuristic that extends a walk one contig at a time, based on a score that accounts for similarity between the contig read coverage and the mean coverage of the currently assembled walk. Putative plasmid bins are selected from the network using graph partitioning algorithms. HyAsP (Müller and Chauve 2019) uses a greedy walk-building heuristic that aims to iteratively extract walks from the assembly graph. The algorithm relies on a walk-extension objective function defined in terms of coverage consistency, Guanine-Cytosine (GC) content consistency, and high plasmid gene density (from a database of reference plasmid genes) of contigs included in the walk. PlasBin (Mane et al. 2022) extends the approach used in HyAsP into a Mixed Integer Linear Programming (MILP) formulation that defines plasmid bins as connected subgraphs of the assembly graph that optimize an objective function similar to the HyAsP one.

In this work, we introduce a new optimization algorithm for the plasmid binning problem. The main novelty of our work is to state the problem as a network flow problem, where the flow accounts for the expected uniform coverage of the sequenced plasmid, while also considering the GC content and the density of reference plasmid genes in a plasmid bin. Our work improves upon PlasBin in several ways, including a method for scoring GC content based on a probabilistic model. We compare our method PlasBin-flow against PlasBin, HyAsP, plasmidSPAdes, MOB-recon, and gplas on a dataset of 133 bacterial samples.

## 2 Method

Here we present PlasBin-flow, a network flow-based MILP formulation for the plasmid binning problem. It extracts putative plasmid bins as connected components of uniform coverage from an assembly graph. PlasBin-flow is a hybrid method that relies on a reference database of closed genome assemblies including plasmids.

### 2.1 Input: Contigs and the assembly graph

The input of the PlasBin-flow consists of the contigs and the assembly graph from a short-read assembly of a bacterial isolate. In this work, we use Unicycler (Wick et al. 2017) to generate the assembly, but any other assembler that provides an assembly graph (such as PlasmidSPAdes (Antipov et al. 2016) or SKESA (Souvorov et al. 2018)) can be used, as PlasBin-flow does not depend on a specific assembler.

The set of all contigs in the short-read assembly is denoted by *C*. Every contig c∈C has two extremeties: head $c_{h}$ and tail $c_{t}$. Pairs of contigs potentially adjacent in the genome sequence are connected via edges of the assembly graph, each edge linking two specific contig extremities. We denote the set of edges in the assembly graph by *E*. Each undirected edge e∈E connects two extremities $c_{u}$ and $d_{v}$ ($c_{u}$ and $d_{v}$ being either a head or a tail of contigs *c* and *d*, respectively) and is represented as an unordered pair $\left\{c_{u},d_{v}\right\}$.

The PlasBin-flow input also contains a reference database of plasmid genes. In a practical application, the reference database would contain all known plasmid genes; however, since an exact match to the reference database would indicate a strong determination of the contig origin, we have taken care in our experiments to withdraw information from samples used for testing from the database used (see Section 3.1 for further description).

#### 2.1.1 Contig features

PlasBin-flow considers several features associated to a contig *c*, namely its GC content $gc_{c}$, length $\ell_{c}$, sequencing coverage $rc_{c}$, and plasmid gene density $gd_{c}$. The sequencing coverage is the normalized coverage provided by Unicycler for each contig, i.e. the average base coverage of the contig normalized by the median coverage of the contigs in the whole assembly; chromosomal contigs are thus expected to have a normalized coverage close to 1. To obtain the plasmid gene density, we map each contig against the reference database of plasmid genes using blastn (version 2.6.0) (Camacho et al. 2009), discarding any match with identity below 95% or a match that covers ˂95% of a gene. The plasmid gene density $gd_{c}$ is the fraction of the sequence length of contig *c* covered by these matches.

Contigs passing certain thresholds related to length and gene density are referred to as seed contigs. These contigs are more likely to be part of a plasmid than others. We describe how to determine corresponding thresholds using the reference database in Section 2.4. The set of all seed contigs is denoted as $C_{s}$, and we also use a boolean feature $s_{c}=[c\in C_{s}]$.

### 2.2 PlasBin-flow overview

PlasBin-flow works iteratively, identifying one plasmid bin in each iteration by solving a MILP, described in Section 2.3. In each iteration, the identified bin is required to contain at least one seed contig. After each iteration, PlasBin-flow updates the assembly graph (contigs, edges, and contig read coverage) to account for the removal of the plasmid bin. The modified assembly graph is then used as input for the next iteration. This process is repeated until the modified assembly graph does not contain any seed contig.

PlasBin-flow defines a plasmid bin as a connected subgraph of a network defined from the assembly graph, while enforcing that this subgraph contains at least one seed contig and assigning to this subgraph (i) a flow value defined in terms of the coverage of contigs it contains, that serves as a proxy for the copy number of the plasmid (bin) defined by the subgraph and (ii) a GC content value. This subgraph is chosen as the one optimizing an objective function defined as a linear combination of several terms: (i) the flow value, (ii) a GC content term penalizing contigs whose GC content does not agree with the GC content assigned to the subgraph, and (iii) a plasmid gene density term penalizing contigs with a low plasmid gene density. An important and novel feature of PlasBin-flow is that it assigns a multiplicity to each contig in a plasmid bin, with repeated contigs having multiplicity ˃1.

The GC content assigned to a solution is defined as a discrete category, each of the *k* categories corresponding to a GC content interval. This set *K* of *k* intervals is pre-defined through a preliminary analysis of the reference database. For each contig *c* and each GC content interval b∈K, we pre-compute a GC content penalty $gc\_pen_{c,b}$ defined in terms of the likelihood that a sequence of length $\ell_{c}$ and GC content $gc_{c}$ would be observed in a plasmid whose GC content is in interval *b*. We provide a detailed description of $gc\_pen_{c,b}$ in Section 2.5.

### 2.3 MILP formulation

#### 2.3.1 Network and flow

The MILP implemented in PlasBin-flow takes as an input network *N* obtained by modifying the original assembly graph. Using contigs *C* and edges *E* in the undirected assembly graph, we define a directed network $N=(C^{\prime }\cup \{s,t\},E^{\prime }\cup I^{\prime })$. The new set of nodes is composed of $C^{\prime }$, the set of all contig extremities and {s,t}, the source and sink nodes of the network. The new set of edges is composed of two subsets, $E^{\prime }$ and $I^{\prime }$. Set $E^{\prime }$ is obtained by (i) doubling each edge $\{c_{u},d_{v}\}\in E$ into two directed edges $\left(c_{u},d_{v}\right)$ and $\left(d_{v},c_{u}\right)$, (ii) adding edges $\left(s,c_{t}\right)$ and $\left(s,c_{h}\right)$ for every seed contig *c* and (iii) adding edge $\left(d_{h},t\right)$ and $\left(d_{t},t\right)$ for every contig *d* (regardless of its seed status). Set $I^{\prime }$ is composed of directed edges that connect extremities within each contig, i.e. for each contig c∈C, $I^{\prime }$ contains edges $\left(c_{h},c_{t}\right)$ and $\left(c_{t},c_{h}\right)$.

The choice of the subgraph, whose contigs will form a plasmid bin, is guided by a flow through the network. Expanding the set of undirected edges *E* to the set of directed edges $E^{\prime }$ is necessary to allow the flow to account for the orientation of contigs in the flow computed by the MILP. Moreover, every edge $e=(c_{u},d_{v})$ has a capacity $cap_{e}=\min\{rc_{c},rc_{d}\}$ that provides an upper-bound to the total amount of flow that can go through *e*, while if *e* links a contig extremity $c_{u}$ to the source *s* or sink *t*, its capacity is $rc_{c}$. We define a flow as a map $f:E^{\prime }\to R$ that satisfies the following constraints:

- Source and sink constraints*.* Exactly one edge outgoing from *s* and incoming to *t* has a non-zero flow value.
- Capacity constraints for edges: The flow through an edge cannot exceed its capacity.
- Conservation constraints: The cumulative flow into a contig extremity (sum of the flow of the edges from $E^{\prime }$ into this contig extremity) should be equal to the cumulative flow out of the other extremity of the same contig (sum of the flow of the edges from $E^{\prime }$ out of this contig extremity).
- Capacity constraints for nodes: The cumulative flow into and out of a contig extremity cannot exceed the read coverage of the contig.

The value of the flow *F* is thus equal to the quantity flowing out of *s*. By the conservation constraints, this is also equal to the quantity flowing into *t*. The flow value can be considered as a proxy for the copy number of the plasmid represented by the bin defined by the subgraph induced by the non-zero flow edges. A contig from this subgraph can represent a sequence that has been repeated in the plasmid. To account for such instances, the flow through a contig can be higher than the overall flow *F*. However, this does not imply that the flow through a contig will be an integer multiple of *F*.

#### 2.3.2 Decision variables

The MILP decision variables define a solution to the optimal plasmid bin problem, composed of a subgraph of *N*, GC content value associated to it and a flow value. To each element of *N*, we wish to associate a decision binary variable to indicate whether it is part of the solution subgraph $N_{p}$ associated with a plasmid bin *p*. $N_{p}$ is defined by a set of edges from $E^{\prime }$, a set of contig extremities from $C^{\prime }$ and a set contig edges from $I^{\prime }$. If an extremity $c_{h}$ (resp.$c_{t}$) belongs to $N_{p}$, so should the other extremity $c_{t}$ (resp. $c_{h}$). Additionally, at least one internal edge $\left(c_{h},c_{t}\right)$ or $\left(c_{t},c_{h}\right)$ has to be selected to facilitate the flow within contig *c*. Thus, instead of having a variable each associated with extremities $c_{h}$ and $c_{t}$ as well as internal edges $\left(c_{h},c_{t}\right)$ and $\left(c_{t},c_{h}\right)$, it suffices to have a variable $x_{c}$ associated with contig *c*. Thus, $x_{c}=1$ will mean that both extremities of *c* and at least one internal edge of *c* will be part of $N_{p}$. We also associate binary decision variables to each edge $e\in E^{\prime }$, denoted respectively $y_{e}$, indicating whether it is a part of the solution subgraph $N_{p}$. We call the edges and contigs included in the solution active.

We associate to every edge $e\in E^{\prime }$ a continuous decision variable $f_{e}$ that encodes the quantity f(e) flowing through the edge. The overall flow value (flowing out of the source *s* and into the sink *t*) is encoded by a continuous decision variable *F*. The flow *F* also helps in determining the multiplicity for each contig in the plasmid bin defined by a solution. Let $c_{u}^{+}$ be the set of edges incoming into extremity $c_{u}$ and $c_{u}^{-}$ be the set of edges outgoing from $c_{u}$. The multiplicity of a contig $m_{c}$ in the plasmid bin is related to the flow value as $m_{c}=\sum_{e\in c_{h}^{+}\cup c_{t}^{+}}f_{e}/F$.

The GC content of the solution subgraph is chosen as one of the *k* pre-defined intervals and is encoded by binary decision variables: for every pre-defined GC content interval b∈K, the decision variable $GC_{b}$ is assigned value 1 if *b* is the chosen GC content interval and 0 otherwise.

The total number of decision variables defining a solution to the plasmid bin finding problem (subgraph, associated flow, and GC content) is thus $O(|C|+|E^{\prime }|+|K|)$. Note that the subgraph of *N* defined as above may not be connected. However, we aim to define a plasmid bin by a connected subgraph. We describe later how we handle this problem through delayed constraints generation.

#### 2.3.3 Objective function

We formulate the MILP as a maximization problem with objective function F+GC+GD. Here, *F* represents the overall flow value from *s* to *t*, *GC* is a penalty for active contigs whose GC content differs significantly from the overall bin GC content and *GD* is a term penalizing inclusion of contigs with low plasmid gene density and rewarding contigs with a high gene density. In particular, and


$$GC=\sum_{c\in C,b\in K}-(gc\_pen_{c,b})\cdot x_{c}\cdot GC_{b},$$

$$GD=\sum_{c\in C}\left(gd_{c}-0.5\right)\cdot x_{c}.$$

Note that the term *GC* is not linear as it involves the product of two binary decision variables. Such a product can be linearized by addition of a single auxiliary binary decision variable and three associated linear constraints (see Supplementary Material Section S1). The gene density in the objective function is used in a similar manner as probability. Thus, the MILP is discouraged from choosing contigs with gene density ˂0.5 as part of the solution.

The rationale for this objective function is as follows: by maximizing the flow value *F*, we aim to identify a high copy-number plasmid i.e. more likely to be reliable. The two penalty terms aim to assign to the plasmid bin a GC content that reflects the GC content of the selected contigs (we refer to Section 2.5 for a more detailed discussion) and prefer contigs that have a higher plasmid genes density and are thus more likely to originate from actual plasmids. Our approach that seeks a plasmid bin through optimization improves upon greedy heuristics (such as HyAsP) in that it will include contigs that impact negatively the objective function if this inclusion allows to connect to other contigs whose positive contribution to the objective function will be beneficial overall. In contrast, HyAsP, being based on a greedy walk extension approach, may stop a walk extension simply because all neighboring contigs have a negative impact on the objective function. Advantage of the optimization approach over this greedy heuristic is illustrated by the results shown in Section 3.3, Tables 1 and 2.

**Table 1.** Comparison of tool accuracy on 41 bacterial isolates.^a^

	Basepair level			Contig level		
	(weighted)			(unweighted)		
Tool	Precision	Recall	F1	Precision	Recall	F1
PlasBin-flow	0.75	0.71	0.67	0.79	0.63	0.64
PlasBin	0.59	0.57	0.55	0.59	0.56	0.54
HyAsP	0.74	0.48	0.55	0.74	0.40	0.48
plasmidSPAdes	0.54	0.68	0.54	0.47	0.70	0.50
MOB-recon	0.83	**0.91**	**0.85**	**0.81**	**0.83**	**0.81**
gplas	**0.84**	0.74	0.76	0.79	0.58	0.64

a The table shows the mean values of precision, recall, and F1 statistics, considering only contigs of length at least 1000 bp, over 41 testing samples from *E. coli*, *E. faecium*, and *K. pneumoniae*, the species supported by gplas.

Bold values indicate the highest values in the respective columns.

**Table 2.** Comparison of tool accuracy on 25 bacterial isolates.^a^

	Basepair level			Contig level		
	(weighted)			(unweighted)		
Tool	Precision	Recall	F1	Precision	Recall	F1
PlasBin-flow	**0.98**	0.90	**0.93**	**0.96**	0.66	**0.76**
PlasBin	0.72	0.57	0.57	0.64	0.58	0.58
HyAsP	0.96	0.85	0.89	0.90	**0.70**	**0.76**
plasmidSPAdes	0.47	0.61	0.49	0.35	0.54	0.34
MOB-recon	0.92	**0.97**	0.91	0.94	**0.70**	**0.76**

a The table shows mean values of precision, recall, and F1 statistics for the remaining 25 testing samples from species not supported by gplas.

Bold values indicate the highest values in the respective columns.

#### 2.3.4 Constraints

We now describe the constraints used in the formulation:

1. Exactly one edge out of *s* is part of the solution:
  $$\sum_{e=(s,c_{u}),c\in C_{s},u\in \{h,t\}}y_{e}=1.$$
  This also ensures that every solution contains at least one seed contig.
2. The flow through an edge *e* is non-zero only if *e* was selected in the solution subgraph and cannot exceed its capacity:
  $$f_{e}\leq cap_{e}\cdot y_{e}.$$
3. For any contig *c*, the cumulative flow through *c* cannot exceed its read coverage:
  $$\sum_{e\in c_{h}^{+}\cup c_{t}^{+}}f_{e}\leq rc_{c}.$$
4. For any contig *c*, the cumulative flow into $c_{h}$ should be equal to the cumulative flow out of $c_{t}$ and conversely:
  $$\sum_{e\in c_{h}^{+}}f_{e}=\sum_{e\in c_{t}^{-}}f_{e},\;\sum_{e\in c_{t}^{+}}f_{e}=\sum_{e\in c_{h}^{-}}f_{e}.$$
5. Flow value *F* should be equal to the flow out of *s* and into *t*:
  $$F=\sum_{e=(s,v)}f_{e},\;F=\sum_{e=(v,t)}f_{e},$$
6. If an edge $e=(c_{u},d_{v}),c\in C,u,v\in \{h,t\}$ is in the solution ($y_{e}=1$), then the contigs *c* and *d* should also be in the solution. Note that the converse need not be true. In other words, contigs *c* and *d* may be part of the solution due to other edges. In that case, $y_{e}$ should be 0. To implement this, we use the following constraints:
  $$y_{e}\leq \min\{x_{c},x_{d}\}$$
7. A contig *c* is active (selected in the solution) if and only if at least one edge incident to one of its extremities is active.
  $$x_{c}=\min\{1,\sum_{e\in c_{h}^{+}\cup c_{t}^{+}}y_{e}\}$$
8. Each active edge has flow at least *F*. To implement this condition, we introduce an auxiliary variable $F_{e}$
  $$F_{e}=F\cdot y_{e}.$$
  We further add the constraint:
  $$F_{e}\leq f_{e}$$

Thus, if there is no flow through edge *e*, $F_{e}$ is forced to be 0 and in turn $y_{e}=0$. To handle the fact that $F_{e}$ is the product of a binary variable and a continuous variable, we use the same approach as for the *GC* term of the objective function.

#### 2.3.5 Ensuring connectivity

A solution that satisfies the above constraints may not be connected. It will contain a connected component with *s* and *t*, but may also contain other components disconnected from *s* and *t*. Such disconnected components have a closed flow circulating within the component without violating any of the initial constraints.

If the proposed solution contains disconnected components, we mute the edges in the disconnected component. For each edge *e* in the disconnected component, we add a constraint $y_{e}=0$ and run the MILP again. This process is repeated until the MILP returns a solution with a single component connected to *s* and *t*. While in theory, this delayed constraint generation process could involve a large number of iterations, in practice we observe that it requires a small number of iterations. It is possible that this process may force the MILP to overlook solutions containing some of the muted edges even if the solutions have a greater flow. We discuss more effective ways to handle disconnected components in Section 4.

### 2.4 Determining seeds

Similarly to HyAsP and PlasBin, PlasBin-flow enforces that every plasmid bin contains at least one seed contig under the assumption that such contigs were identified as likely to belong to a plasmid due to their sequence features (length and plasmid gene density). We used the reference database for which closed assemblies with annotated plasmids were available, to obtain the thresholds determining seed contigs. Using Unicycler, we assembled the reads datasets for each sample in the reference database. We explored a grid of pairs of values (ℓ,gd), respectively, for the contig length and plasmid gene density, with ℓ ranging from 50 to 5000 bp in increments of 50 and *gd* ranging from 0.01 to 1 in increments of 0.01. For each pair (ℓ,gd) we considered as a seed every contig *c* such that $\ell_{c}\geq \ell ,gd_{c}\geq gd$; then mapping the contigs onto the closed assemblies of the reference dataset, for which plasmids are known, we counted (i) the number *SP* of plasmids onto which at least one seed contig was mapped to (seeded plasmids), and (ii) the number *NPS* of seed contigs that mapped to the chromosome (non-plasmid seeds). We then determined the pair of thresholds that maximized the expression SP−NPS and used them as thresholds defining seed contigs in our experiments. The pair of thresholds thus obtained was (2650,0.58).

### 2.5 Determining GC content intervals and penalties

For each contig *c*, we associate a penalty relating the observed GC content of *c* to the GC content interval b∈K associated to a solution to the MILP. This penalty is based on a probabilistic model in which we can compute the likelihood that a contig of a particular length and GC content will belong to a plasmid whose GC content is in a particular interval *b*. In this section, we first define the model and describe how we define the GC content penalty term. Subsequently, we describe the set of intervals *K* used in our experiments.

#### 2.5.1 Probabilistic model

If a given contig *c* of length ℓ has GC content x∈(0,1), we model the observed number *n* of GC nucleotides within this contig by the binomial distribution with parameters *x* and ℓ. However, GC content within a plasmid may fluctuate for various biological reasons, and therefore we model contig-level GC content *x* as a random variable sampled from a beta distribution with parameters α=pm and β=(1−p)m, where p∈(0,1) is the true GC content of the whole plasmid and m>0 is a fixed parameter. This results in *n* being sampled from the beta-binomial distribution with parameters ℓ, α and β; namely, $\mathrm{Pr}(n|p,\ell )=\left(\begin{array}{c} \ell \\ n \end{array}\right)B(n+\alpha ,\ell -n+\beta )/B(\alpha ,\beta )$ where *B* is the beta function. Values α and β represent pseudocounts added to the observed counts of GC and non-GC nucleotides, respectively. We use m=10 for the overall pseudocount.

In our scenario, *p* is an unknown value coming from GC content interval $b=[p_{s},p_{e}]$, and we assume a uniform prior on *p*. The overall probability of *b* being the true source interval for observed count *n* is thus $\int_{p_{s}}^{p_{e}}Pr(p|n,\ell )dp$, where Pr(p|n,ℓ)∝Pr(n|p,ℓ)⋅Pr(p) is obtained by the Bayes theorem. We normalize these probabilities over all intervals in *K* to obtain the likelihood $gc\_\mathrm{pro}b_{c,b}$ that contig *c* originates from a molecule with GC content in interval *b*.

#### 2.5.2 Penalty term

For contig *c* and interval *b*, the MILP uses penalty $gc\_pen_{c,b}$, which is incurred if *c* is part of a plasmid whose GC content belongs to *b*. This penalty is computed as


$$gc\_pen_{c,b}=\underset{b^{\prime }\in K}{\max}\{gc\_\mathrm{pro}b_{c,b^{\prime }}\}-gc\_\mathrm{pro}b_{c,b}.$$

The penalty is thus zero for the interval *b* where the likelihood of *c* achieves maximum. The penalty increases with the gap between the proposed GC content interval *b* and the most likely interval for contig *c*. For very short contigs, probabilities of different GC content intervals are closer to each other, and thus the resulting penalties are smaller compared to longer contigs.

#### 2.5.3 Pre-defining GC content intervals

The GC content intervals considered were pre-determined prior to running experiments based on an analysis of the reference dataset. The GC content of most plasmids and chromosomes in the reference dataset was observed to be between 0.4 and 0.6. The interval [0.4, 0.6] was subdivided into 4 equal intervals resulting in k=6 intervals K={[0,0.4],[0.4,0.45],[0.45,0.5],[0.5,0.55],[0.55,0.6],[0.6,1]}.

## 3 Experimental results

### 3.1 Datasets and tools

We evaluated the following methods: our new tool PlasBin-flow, HyAsP (Müller and Chauve 2019), MOB-recon (Robertson and Nash 2018), plasmidSPAdes (Antipov et al. 2016), and gplas (Arredondo-Alonso et al. 2020). PlasmidSPAdes and gplas are *de-novo* binning methods, while MOB-recon, HyASP, and PlasBin-flow rely on a reference database of plasmid genes.

Our dataset consists of 133 bacterial genomes and 377 plasmids from a collection of real bacterial isolates with closed genome assemblies compiled by Robertson and Nash (2018). To simulate the use of a realistic reference database, we split our data into a reference set and a test set; the samples released before 19 December 2015 were used to build the reference database and those released after that date formed the test set. The reference set consists of 67 bacterial isolates with 230 plasmids containing 10 685 plasmid genes.

The remaining test set consists of 66 samples with 147 plasmids. For the test set, Illumina sequencing data was re-assembled using Unicycler to provide contigs and assembly graphs, except in case of plasmidSPAdes. PlasmidSPAdes takes raw reads as input and constructs its own assembly graph using the SPAdes assembler. Gplas requires the use of mlplasmids (Arredondo-Alonso et al. 2018) to compute the probability of contigs to originate from a plasmid. Since mlplasmids is a species-specific tool, it currently supports only four species, three of which feature in our test dataset (*Escherichia coli*, *Enterococcus faecium*, and *Klebsiella pneumoniae*). From the test dataset, 41 samples with 88 plasmids belong to these three species.

### 3.2 Evaluation metrics

To evaluate predictions of individual tools on a particular testing sample, we need to compare them to the ground truth determined from annotated finished assembly of the sample as given by Robertson and Nash (2018). We consider two sets of measures: weighted or base-pair level statistics, which consider accuracy of bin predictions weighted by the lengths of individual contigs (effectively counting how many base pairs were involved in correct predictions), and unweighted or contig-level statistics, which only consider accuracy of predictions for individual contigs, disregarding contig lengths.

Each short-read contig in the isolate assembly is mapped against the plasmids from the corresponding finished assembly (ground truth) using BLAST+ (Camacho et al. 2009). Matches that span ˂95% of the contig length are discarded. Thus for each bin *u* predicted by a particular tool, we have a set of contigs $P_{u}$ included in the bin *u*, and for each ground-truth plasmid *v*, we have a set of contigs $T_{v}$ that match (via BLAST+) to the ground-truth plasmid *v*. It should be noted that plasmidSPAdes is run on a different set of contigs than the other five methods. Thus, the sets of contigs representing the ground truth plasmids are different in case of plasmidSPAdes.

For the two sets of contigs $P_{u}$ and $T_{v}$, we define $\text{overlap}(P_{u},T_{v})$ as either $\left|P_{u}\cap T_{v}\right|$ (in case of unweighted statistics) or the cumulative size in base pairs of all contigs in $P_{u}\cap T_{v}$ (in case of weighted statistics). Similarly, we define size(X) for a set of contigs *X* as either |X| (in case of unweighted statistics) or the cumulative size in base pairs of all contigs in *X* (in case of weighted statistics).

In order to compute the precision, we assign to each predicted plasmid bin *v* one of the true plasmids f(v) such that $\text{overlap}(P_{v},T_{f(v)})$ is maximized. The precision is then computed as $\text{overlap}(P_{v},T_{f(v)})/\text{size}(P_{v})$. Similarly, to compute the recall, we assign to each true plasmid *u* one of the predicted plasmid bins g(u) such that $\text{overlap}(P_{g(u)},T_{u})$ is maximized. The recall is then computed as $\text{overlap}(P_{g(u)},T_{u})/\text{size}(T_{u})$.

For a sample with a set P of predicted plasmid bins and T of true plasmids, the mean precision for the sample is then computed as:


$$\frac{\sum_{v\in P}\text{overlap}(P_{v},T_{f(v)})}{\sum_{v\in P}\text{size}(P_{v})}$$

The mean recall for the sample is computed as:


$$\frac{\sum_{u\in T}\text{overlap}(P_{g(u)},T_{u})}{\sum_{u\in T}\text{size}(T_{u})}$$

The F1 measure for a sample is computed as a harmonic mean of mean precision and mean recall for that sample.

### 3.3 Results

For the set of 41 samples from species supported by gplas, we only consider contigs of length at least 1000 bp for our evaluation in order to be fair to gplas that has been evaluated with such a restriction in (Arredondo-Alonso et al. 2020). All contigs, irrespective of length, are considered for the evaluation of the remaining 25 samples.

**Figure 1.**
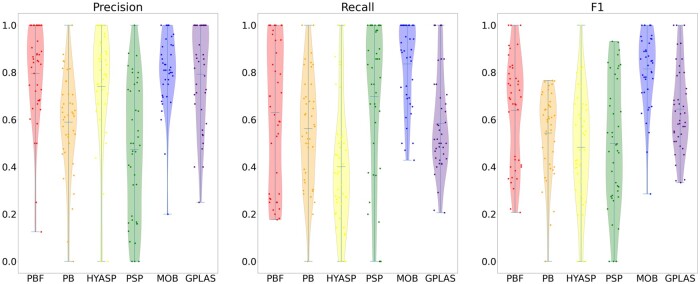
Distribution of the unweighted precision, recall, and F1-score statistics for samples from three species supported by gplas.

**Figure 2.**
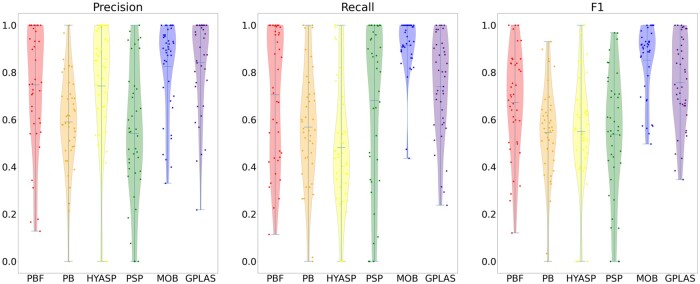
Distribution of the weighted precision, recall, and F1-score statistics for samples from three species supported by gplas.

Table 1 shows both unweighted and weighted accuracy measures (mean precision, mean recall, and mean F1) for all considered tools averaged over the 41 testing samples, whereas Figs 1 and 2 show the full distribution of these measures. We can observe that in the case of weighted measures, that consider only contigs of length at least 1000 bp, the accuracy of PlasBin-flow lags behind gplas as well as MOB-recon, which ranks as the best tool. PlasBin-flow show significant improvement over its HyAsP, PlasBin. and plasmidSPAdes and a comparable recall to gplas.

Unweighted (contig level) accuracy is a challenging measure, since short contigs have a higher contribution to these measures. Depending on the exact down-stream application, correct binning of shorter contigs may or may not be important. Shorter contigs are more difficult to classify and bin because corresponding sequence-based statistics have a higher variance and homology-based statistics are often non-informative. When considering unweighted accuracy measures, PlasBin-flow shows higher recall than gplas, which is especially selective in the choice of contigs. PlasmidSPAdes also shows high recall, but it suffers from many false predictions (low precision). MOB-suite performs the best overall.

**Figure 3.**
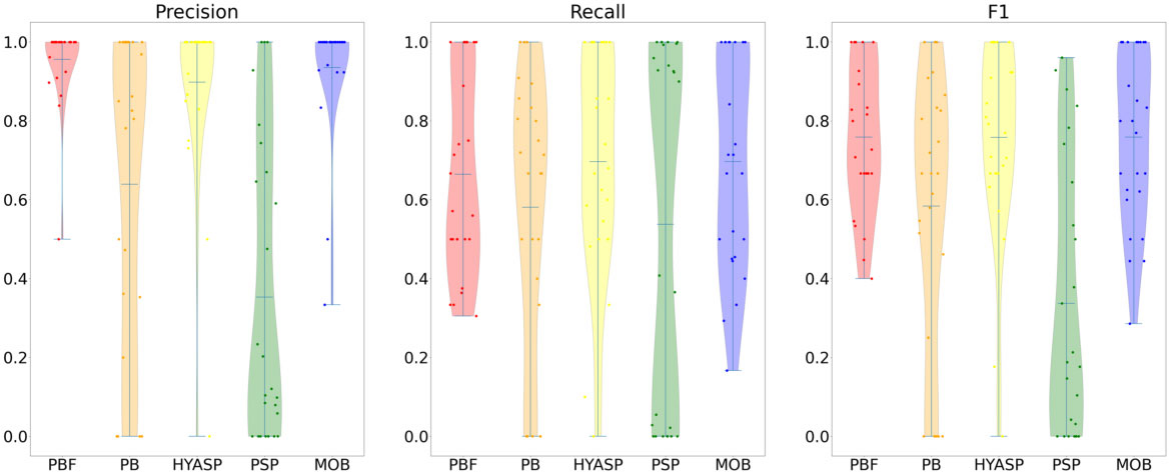
Distribution of the unweighted precision, recall, and F1-score statistics for remaining 25 samples.

**Figure 4.**
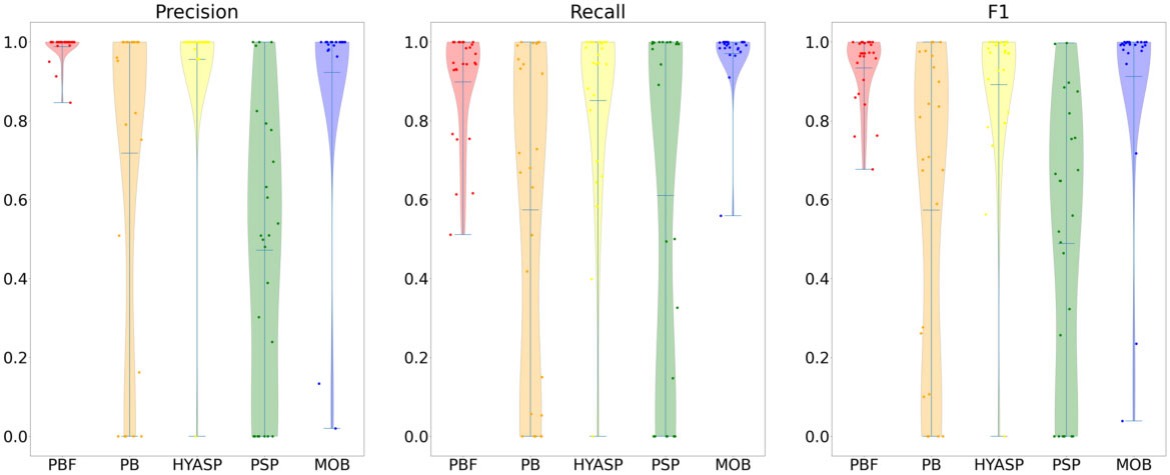
Distribution of the weighted precision, recall, and F1-score statistics for remaining 25 samples.

Table 2 compares both unweighted and weighted accuracy measures for the 25 testing samples that could not be analysed with gplas because they are from unsupported species. Unlike in the analysis of the first 41 samples, we consider all contigs in the evaluation as we do not evaluate gplas on these 25 samples. Figures 3 and 4 show the full distribution of these measures. In the case of weighted accuracy measures, PlasBin-flow shows the best accuracy, but in terms of recall where it ranks second-best behind MOB-recon but shows a better combined F1 accuracy. A similar trend is observed for unweighted accuracy measures.

The performance of PlasBin-flow shows a marked improvement on the latter 25 samples. Compared to other methods, PlasBin-flow is able to leverage the information from the assembly graph and as a result, is able to bin short contigs with higher accuracy than other tools. Since the comparison of the 41 samples from the three species supported by gplas has only been done on contigs longer than 1000 bp (due to inability of gplas to classify shorter contigs), this illustrates that one of the main advantages of PlasBin-flow is how it handles short contigs, that are often numerous in a short-read assembly. In fact, when considering all contigs, PlasBin-flow (F1 accuracy 0.55) outperforms MOB-recon (F1 accuracy 0.45) even on the first 41 samples (refer to Supplementary Material Fig. S1).

We provide in Supplementary Material Section S3 additional figures that show the results of the considered tools for various thresholds of contigs minimum length.

### 3.4 Computational footprint

We ran our experiments on a standard laptop computer, with a quad-core processor and 16GB of memory. PlasBin-flow relies on an MILP, that typically requires large computing resources (time and memory), however, we could observe that PlasBin-flow has a very reasonable computational footprint. In terms of median and mean running times, the method was comparable to other methods such as HyAsP, MOB-recon, and gplas. Excluding a handful of samples that required around 2 hours of computation time, PlasBin-flow usually completed within a few minutes. Over all considered samples, PlasBin-flow had a maximum running time close to 2 h, with a memory footprint close to 6 GB (maximum 10 GB). Running time details are provided in Table 3.

**Table 3.** Running time statistics.^a^

Tool	Minimum	Maximum	Median	Mean
PlasBin-flow	5	120	6	6
PlasBin	5	473	22	117
HyAsP	1	19	3	4
MOB-recon	3	15	5	8
gplas	2	12	3	4
plasmidSPAdes	4	363	16	43

a Median, mean, minimum, and maximum running time (in minutes) recorded over the 66 test samples.

## 4 Conclusion

In this work, we presented PlasBin-flow, an MILP algorithm that uses network flows for grouping contigs into putative plasmid bins. We compared the results of PlasBin-flow against other state-of-the-art plasmid binning methods: HyAsP, plasmidSPAdes, MOB-recon, and gplas using two sets of metrics: weighted (basepair level) and unweighted (contig level). In both cases, PlasBin-flow consistently performed reasonably well in precision statistics. For samples belonging to *E. coli*, *E. faecium*, and *K. pneumoniae*, PlasBin-flow ranked behind MOB-recon and gplas in the average F1 scores. Gplas in particular had a distinct advantage over PlasBin-flow due to its use of species specific training sets. The performance of PlasBin-flow was markedly better on samples belonging to other species. PlasBin-flow was especially useful in binning contigs of short length, which are typically difficult to bin correctly.

The objective function in PlasBin-flow is significantly changed from that used in PlasBin. The uniformity of %GC content along the contigs in a putative plasmid bin is achieved using a probabilistic model. The use of %GC content penalties discourages the choice of contigs disparate %GC content to be selected in the same bin. Another salient features of this method are the use of flow values as a proxy for read coverage used in a solution. The conditions necessary to maintain a consistent flow ensure the uniform read coverage in a plasmid bin without the need to explicitly compute the mean read coverage of the bin. The objective function used in our experiments assigned the same weight to the three terms it contains. This was a choice guided by a limited exploration of possible weights for each term (Supplementary Material Section S2). However, given the importance of the tuning dataset, e.g. to obtain the gene density of contigs, a different choice might be relevant for other datasets, that should result from exploring a grid of possible weights.

In some instances, PlasBin-flow shows low recall. There are two possible explanations for low recall: misidentification and misbinning. In case of misidentification, PlasBin-flow may have failed to identify the contigs belonging to a true plasmid as part of any plasmid bin, e.g. due to the low gene density of a contig. On the other hand, misbinning occurs when a true plasmid is split into multiple bins output by PlasBin-flow. As a result, even if PlasBin-flow manages to identify relevant plasmid contigs, the recall statistics will only consider contigs placed in one of the bins, the contigs in other bins will not contribute toward recall. We further discuss splitting bins as a cause of low recall in (Supplementary Material Section S4).

Through an exploration of the results obtained on our test dataset, we have determined that splitting of bins often occurs when groups of contigs belonging to the same plasmid are connected by a bridge of contigs with low plasmid gene densities. In future work, we will investigate possible remedies for this problem, including: (i) merging bins with similar GC content and coverage depth in post-processing; (ii) modifying the optimization function to provide positive rewards from more sources (at present, only contigs with high plasmid gene densities bring positive rewards); and (iii) building a more comprehensive database of plasmid genes. We expect such changes to further improve overall performance of PlasBin-flow.

The method currently used for delayed constraint generation in the MILP may in some cases disregard the true optimal solution of the underlying optimization problem. We plan to refine the delayed constraint generation so that the added constraints would prevent flow in the disconnected components, rather than forbidding edges previously involved in disconnected components completely. This will guarantee that the optimal solution to the original optimization problem is found and may further help to prevent unnecessary bin splitting; on the other hand, such modification may require more iterations and may negatively affect the running time.

It should be noted that bins generated by PlasBin-flow might not be walks. Ideally, a plasmid, or plasmid segment, should appear as a walk in the assembly graph. However PlasBin-flow computes connected subgraphs without looking explicitly for walks. The flow through the subgraph representing a bin can be seen as a proxy for a walk; however, the problem of extracting walks from a bin subgraph relates more to the actual assembly of plasmids than to the problem of binning and requires further attention.

Our MILP formulation could also accommodate other contig features in its objective function such as including a probabilistic value from plasmid contig classification methods such as mlplasmids, or accounting for the presence of plasmid-specific genes (replicon genes e.g.). This avenue is among the ones we plan to explore further in order to improve the PlasBin-flow model.

## Supplementary Material

btad250_Supplementary_Data

## Data Availability

PlasBin-Flow is open-source and available at https://github.com/cchauve/PlasBin-flow. The database used in the experiments is also available in the same repository. The accession details for the samples as well as the output of all the methods used in the experiments can be accessed at https://doi.org/10.5281/zenodo.7807303.
