# Supplementary material for: PlasBin-flow: a flow-based MILP algorithm for plasmid contigs binning
Source: Bioinformatics. 2023 Jun 30;39(Suppl 1):i288–96. doi: 10.1093/bioinformatics/btad250 (PMC10311310; doi:10.1093/bioinformatics/btad250)
Supplement: btad250_Supplementary_Data [file btad250_supplementary_data.zip › btad250_Supplementary_Data/Mane.291.sup.1.pdf]

PlasBin-flow: A flow-based MILP algorithm for plasmid contigs binning
Supplementary material

Aniket Mane, Mahsa Faizrahnemoon, Tomáš Vinař, Broňa Brejová, Cedric Chauve

Section 1 provides a detailed description of the PlasBin-flow MILP formulation.
Section 2 provides experimental results obtained by considering different weights for the PlasBin-flow objective function terms.
Section 3 provides additional experimental results for PlasBin-flow and the other plasmid binning tools evaluated in our work. These results focus on the accuracy when considering various thresholds for the minimum length of the contigs considered for evaluation.
Section 4 provides more detailed analysis of the recall performance of PlasBin-flow.

S1. MILP formulation

In this section, we provide the details of the MILP formulation. We first remind the input parameters of the MILP and the associated notations, then introduce the decision variables that are used in the MILP. We then provide the full formulation of the MILP in terms of these variables, followed by a discussion on how we handle non-linear terms in the objective function and constraints.

Parameters. The input of PlasBin-flow consists of the contigs and the assembly graph from a short-read assembly of a bacterial isolate. The set of all contigs in the short-read assembly is denoted by C. Every contig $c \in C$ has two extremeties: head c_h and tail c_t. The set of edges in the assembly graph is denoted by E. Each undirected edge $e \in E$ connects two extremities c_u and d_v (c_u and d_v being either a head or a tail of contigs c and d respectively) and is represented as an unordered pair $\{c_u, d_v\}$. Each contig $c \in C$ has the following features associated with it: GC content gc_c, length ℓ_c, sequencing coverage rc_c and plasmid gene density gd_c. A contig c is assigned *seed* status if it passes certain thresholds on gene density and length.

We also define K non-intersecting GC content intervals such that the union of these intervals is $[0, 1]$. For each contig c and each GC content interval $b \in K$, we compute a *GC content penalty* $gc\_pen_{c,b}$ defined in terms of the likelihood that a sequence of length ℓ_c and GC content gc_c would be observed in a plasmid whose GC content is in interval b.

Using the assembly graph, we define a directed network $N = (C' \cup \{s, t\}, E' \cup I')$. The new set of nodes is composed of C', the set of all contig extremities, and $\{s, t\}$, the source and sink nodes of the network. The new set of edges is composed of two subsets, E' and I'. For each edge $\{c_u, d_v\} \in E$, E' contains two directed edges (c_u, d_v) and (d_v, c_u). E' also contains newly constructed edges (s, c_t) and (s, c_h) for every seed contig c as well as (d_h, t) and (d_t, t) for every contig d. Set I' is composed of directed edges that connect extremities within each contig, i.e. for each contig $c \in C$, I' contains edges (c_h, c_t) and (c_t, c_h). The *capacity* of edge $e = (c_u, d_v)$ is defined by $cap_e = \min\{rc_c, rc_d\}$, while if e links a contig extremity c_u to the source s or sink t, its capacity is rc_c.

Variables. Table 1 contains the descriptions of variables used in the MILP. We refer the reader to the main manuscript for the precise definition of the terminology. It should be noted that it is sufficient to have

**Decision variable**	**Type**	**Description**
x_c	Binary	$x_c = 1$ if contig $c \in p$
y_e	Binary	$y_e = 1$ if edge $e \in p$
GC_b	Binary	$GC_b = 1$ if GC content of p belongs to GC bin b
f_e	Continuous	Flow through directed edge e
F	Continuous	Overall flow out of s
**Aux. Variable**	**Type**	**Description**
$ctg\_GC_{c,b}$	Binary	$ctg\_GC_{c,b} = GC_b \cdot x_c$
F_e	Continuous	$F \cdot y_e$

Table 1: Subscripts c, e, p, b respectively refer to a contig c of the initial assembly graph, a directed edge e of the network N, the subgraph p of N computed by the MILP and a GC bin b.

a variable x_c associated with contig c instead of having variables associated with extremities c_h and c_t as well as internal edges (c_h, c_t) and (c_t, c_h).

MILP formulation.

$$\text{Maximize} \quad F + \sum_{c \in C, b \in K} -(gc\_pen_{c,b}) \cdot x_c \cdot GC_b + \sum_{c \in C} (gd_c - 0.5) \cdot x_c$$

$$\text{subject to:} \quad \sum_{e=(s,c_u), c \in C_s, u \in \{h,t\}} y_e = 1 \tag{1}$$

$$f_e \leq cap_e \cdot y_e \tag{2}$$

$$\sum_{e \in c_h^+ \cup c_t^+} f_e \leq rc_c \tag{3}$$

$$\begin{aligned} \sum_{e \in c_h^+} f_e &= \sum_{e \in c_t^-} f_e \\ \sum_{e \in c_t^+} f_e &= \sum_{e \in c_h^-} f_e \end{aligned} \tag{4}$$

$$\begin{aligned} F &= \sum_{e=(s,v)} f_e \\ F &= \sum_{e=(v,t)} f_e \end{aligned} \tag{5}$$

$$y_e \leq \min\{x_c, x_d\} \tag{6}$$

$$x_c = \min\{1, \sum_{e \in c_h^+ \cup c_t^+} y_e\} \tag{7}$$

$$F_e = F \cdot y_e \tag{8}$$

$$F_e \leq f_e \tag{9}$$

$$\begin{aligned} x_c,\ y_e,\ GC_b,\ ctg\_GC_{c,b} &\in \{0, 1\} \\ F,\ F_e,\ f_e &\geq 0 \end{aligned} \tag{10}$$

Constraints to handle non-linear terms : Our formulation contains some terms that are inherently non-linear. We provide the set of linear constraints used to replace such non-linear terms. For instance, the GC penalty term in the objective function appears as a product of two variables. The fact that at least one of the variables in these product is binary allows us to obtain a suitable linear relaxation. We use a technique of McCormick envelopes to obtain this relaxation [1]. We also explain how we obtain a linear set of constraints to emulate constraints that require computing the minimum of two terms.

- The objective function consists of a term that computes the penalty for contig c when its GC content differs significantly from that of the overall plasmid bin. If a putative plasmid is assigned a GC content bin b, the penalties for the GC content for contig c will be considered only if the contig is part of the putative plasmid. Thus, we have

  $$ctg\_GC_{c,b} = x_c \cdot GC_b \tag{11}$$

  Thus, $ctg\_GC_{c,b} = 1$ only when both x_c and GC_b both take value 1. Here, x_c and GC_b are both binary terms. This allows us to define a set of linear constraints to replace the quadratic term.

  $$ctg\_GC_{c,b} \leq x_c \tag{12}$$
  $$ctg\_GC_{c,b} \leq GC_b \tag{13}$$
  $$ctg\_GC_{c,b} \geq x_c + GC_b - 1 \tag{14}$$

  Thus, if either of x_c or GC_b are 0, $ctg\_GC_{c,b} = 0$. If both are 1, the above constraints ensure that $ctg\_GC_{c,b} = 1$.

  Finally, the following constraint takes care of the fact that the GC content of the solution lies in exactly one GC bin.

  $$\sum_b GC_b = 1 \tag{15}$$

- To implement constraint 9, we wish to compute the overall flow F_e through edge e. However, F_e is a quadratic term as defined in constraint 8. This constraint is meant to ensure that only active edges carry non-zero flow. Once again, the fact that y_e is binary helps us use the following constraints to compute F_e:

  $$F_e \leq U \cdot y_e \tag{16}$$
  $$F_e \leq F \tag{17}$$
  $$F_e \geq F - (1 - y_e) \cdot U \tag{18}$$
  $$F_e \geq 0 \tag{19}$$

  Here, U is an upper bound on the flow. We can use $U = max_{c \in C}(rc_c)$. Thus, if $y_e = 1$ then $F_e = F$, else it is equal to 0.

- Constraint 6 ensures that an edge is part of the solution only if both its contigs to which the extremities belong are part of the solution. To implement this constraint, we simply add linear constraints:

  $$y_e \leq x_c \tag{20}$$
  $$y_e \leq x_d \tag{21}$$

  Note that even if $x_c = x_d = 1$, it is not necessary that $y_e = 1$. But, either $x_c = 0$ or $x_d = 0$ forces $y_e = 0$.

- Constraint 7 ensures that contig c is part of the solution if and only if an active edge is incident to one of its extremities. To implement this constraint, we need to introduce constraints:

$$x_c \leq 1 \tag{22}$$

$$x_c \leq \sum_{e \in c_h^+ \cup c_t^+} y_e \tag{23}$$

However, these two constraints are not enough since x_c can take value 0 even if $\sum_{e \in c_h^+ \cup c_t^+} y_e \geq 1$. So, we add another constraint that forces x_c to be positive if an edge is incident on its contig extremity.

$$x_c \geq \sum_{e \in c_h^+ \cup c_t^+} y_e / M \tag{24}$$

Here, M is the upper bound on the number of edges adjacent on c. Dividing by this upper bound ensures that x_c does not exceed 1.

S2. Results with weighted objective function

The objective function in the current MILP assumes equal weights for each term in the objective function. Our experimental results were obtained with this specific formulation. This section describes limited experimental results with different weighting schemes that motivates this choice on the considered datasets.

The three terms in the objective function correspond to the flow term F, the GC content penalty term GC and lastly, the gene density term GD. We assigned weights α_1, α_2 and α_3 respectively to the above terms, considering for each the values 1 and 2, and exploring all possible combinations aside of all three weights equal to 2, which would be equivalent to the case of all three weights equal to 1. The considered data were the 66 samples of our test data set (see main manuscript).

Table 2 shows the precision, recall and F1 statistics (as defined in the main manuscript) for this experiment. An interesting pattern is observed in the evaluation. For the chosen weight combinations, there was no significant variation in the statistics. However, certain combinations seem to perform well in terms of precision while some others in recall. The original combination of equal weights consistently ranks among the top two in terms of F1-score. Increasing the weight of the flow term (α_1) tends to improve precision. On the other hand, increasing α_3 slightly improves recall but at the expense of precision. Changing α_2 does not display any specific pattern as such.

When the evaluation is restricted to longer contigs, Table 3 indicates that increasing α_1 does not always improve precision. However, it does negatively impact recall. α_3 shows the same pattern as before, improving recall at the cost of precision.

In general, giving higher importance to the flow value tends to result in a lower recall. As expected, increasing the weight for the gene density term encourages the MILP to include more contigs, in turn, resulting in better recall. However, this also seems to affect precision negatively. Overall, the results suggest that the combination of equal weights yields the best results most of the time.

Table 2: **Comparison of accuracy on all 66 bacterial isolates.** The table shows the mean values of precision, recall and F1 statistics, considering all contigs for different weight combinations

Objective function weights	Basepair level (weighted)			Contig level (unweighted)		
$\alpha_1.\alpha_2.\alpha_3$	Precision	Recall	F1	Precision	Recall	F1
1.1.1	**0.840**	0.772	**0.769**	0.848	0.567	0.631
1.1.2	0.799	**0.774**	0.747	0.838	0.570	0.630
1.2.1	0.833	0.767	0.765	0.850	0.562	0.627
1.2.2	0.809	0.768	0.751	0.841	**0.574**	**0.633**
2.1.1	**0.840**	0.753	0.762	**0.854**	0.548	0.616
2.1.2	0.822	0.762	0.755	0.842	0.558	0.621
2.2.1	0.832	0.744	0.753	**0.854**	0.534	0.606

Table 3: **Comparison of accuracy on all 66 bacterial isolates.** The table shows the mean values of precision, recall and F1 statistics, considering only contigs of length at least 1000 bp for different weight combinations

Objective function weights	Basepair level (weighted)			Contig level (unweighted)		
$\alpha_1.\alpha_2.\alpha_3$	Precision	Recall	F1	Precision	Recall	F1
1.1.1	0.838	0.780	**0.773**	0.860	0.667	**0.705**
1.1.2	0.797	**0.781**	0.749	0.845	0.672	0.702
1.2.1	0.832	0.774	0.768	0.860	0.663	0.701
1.2.2	0.808	0.774	0.754	0.848	**0.674**	0.704
2.1.1	**0.839**	0.760	0.765	**0.863**	0.643	0.689
2.1.2	0.820	0.769	0.758	0.850	0.654	0.692
2.2.1	0.831	0.751	0.756	0.860	0.634	0.684

S3. Additional results

We evaluated the results for all the tools considered in the manuscripy with weighted as well as unweighted statistics, considering three cases based on thresholds on the minimum length of contigs accounted for in computing the accurcy statistics.

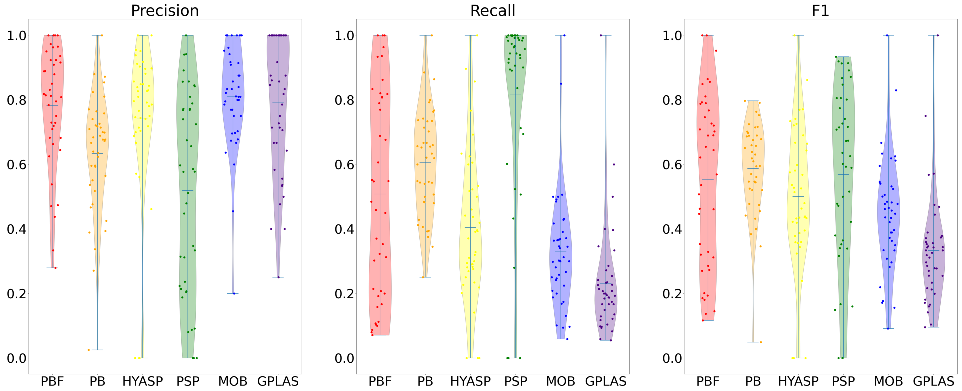

Figure 1: Unweighted statistics for *E. coli*, *E. faecium* and *K. pneumoniae* samples using all contigs

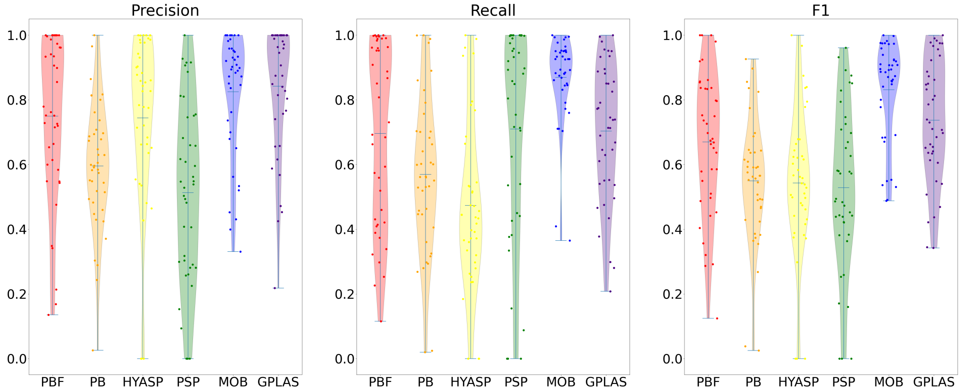

Figure 2: Weighted statistics for *E. coli*, *E. faecium* and *K. pneumoniae* samples using all contigs

From figure 1, we observe that PlasBin-flow generally outperforms other methods in contig level statistics when all contigs are considered for evaluation. Particularly, its recall is much better than MOB-recon for shorter contigs. PlasmidSPAdes is the best in terms of average recall over all samples. However, it lags behind in precision. For the case of base level statistics however, MOB-recon performs the best among all the methods as seen in figure 2. A similar trend is observed when contigs shorter than 100 bp are excluded from the evaluation as shown in figures 3 and 4. Thus, PlasBin-flow is successful in recognizing and correctly binning short contigs.

Figures 5-8 show the statistics for samples not belonging to *E. coli*, *E. faecium* and *K. pneumoniae*. PlasBin-flow, HyAsP and MOB-recon all show comparable statistics with PlasBin-flow generally having an edge in precision and MOB-recon in recall. Overall, PlasBin-flow tends to perform better than other tools.

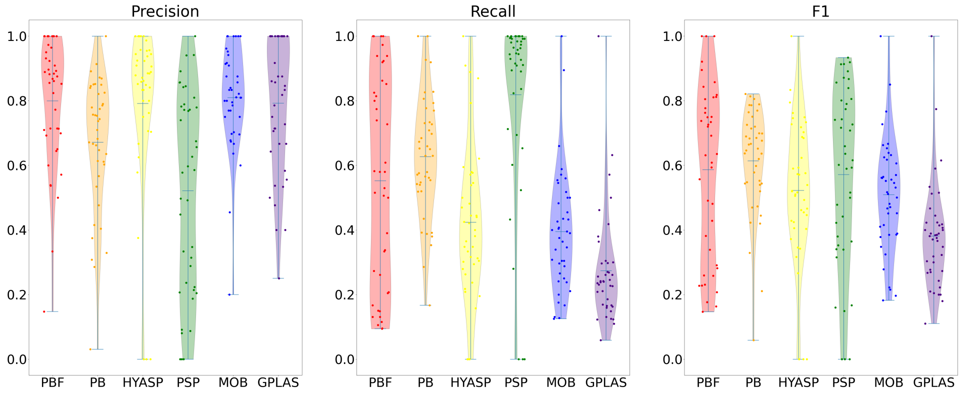

Figure 3: Unweighted statistics for *E. coli*, *E. faecium* and *K. pneumoniae* samples using contigs $\geq$ 100 bp

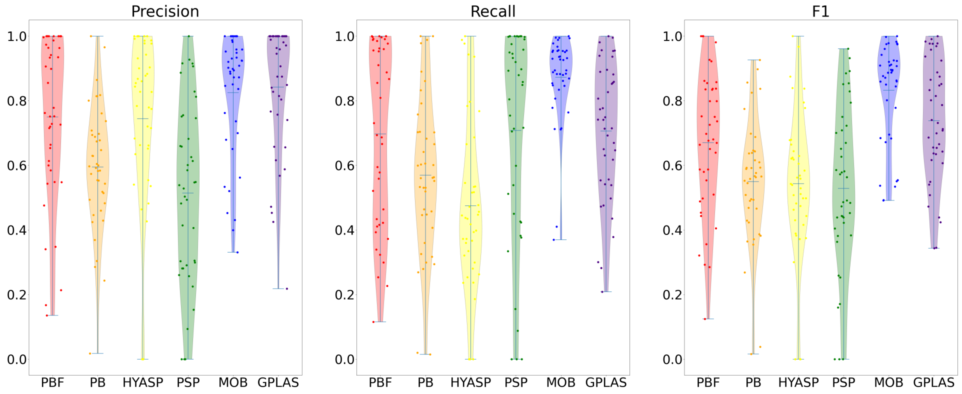

Figure 4: Weighted statistics for *E. coli*, *E. faecium* and *K. pneumoniae* samples using contigs $\geq$ 100 bp

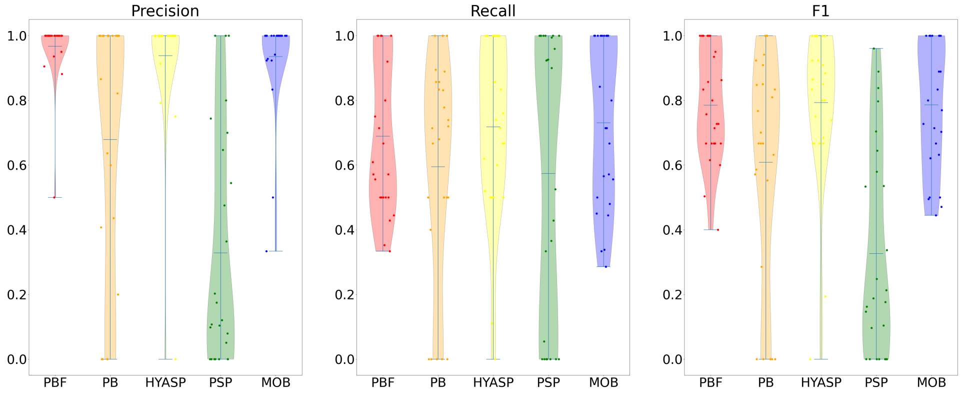

Figure 5: Unweighted statistics for remaining 25 samples using contigs $\geq$ 100 bp

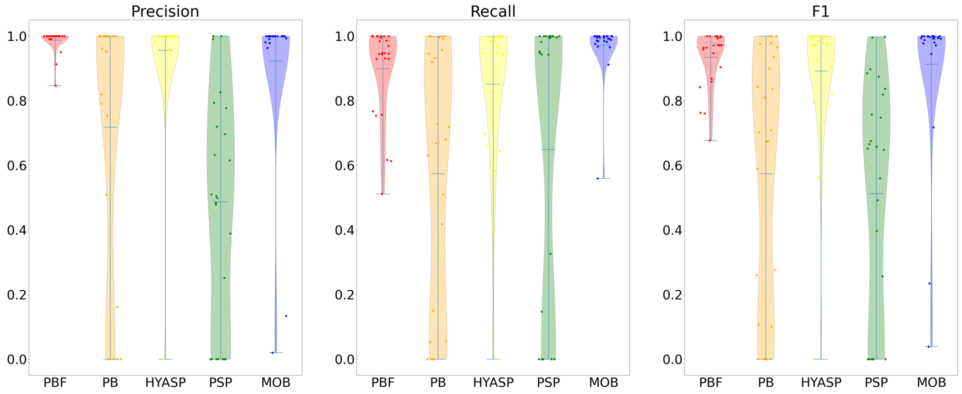

Figure 6: Weighted statistics for remaining 25 samples using contigs $\geq$ 100 bp

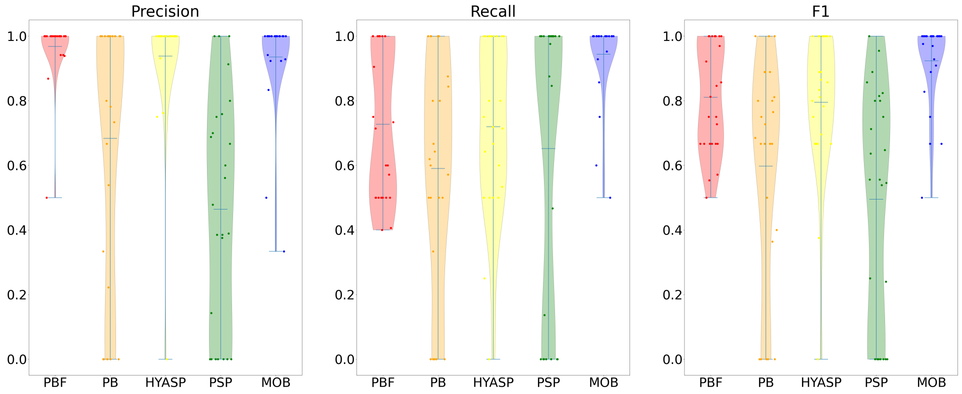

Figure 7: Unweighted statistics for remaining 25 samples using contigs $\geq$ 1000 bp

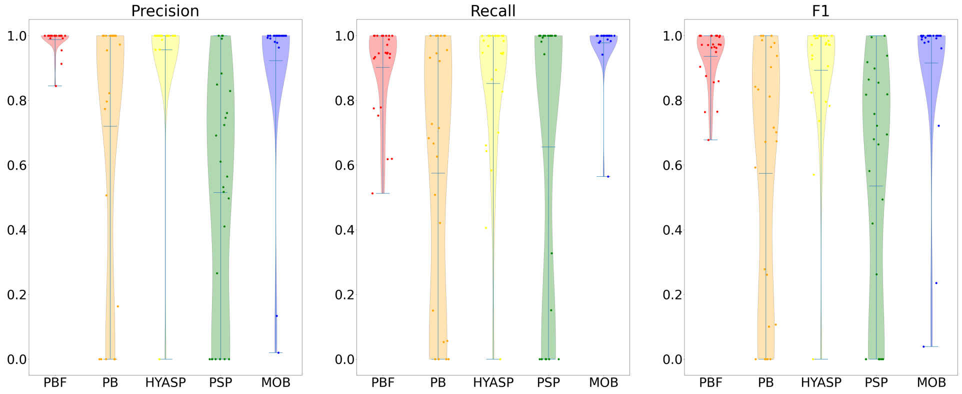

Figure 8: Weighted statistics for remaining 25 samples using contigs $\geq$ 1000 bp

S4. Analyzing issues with low recall

We observed that PlasBin-flow is sometimes outperformed by other tools (mainly MOB-recon) in terms of recall. In particular, PlasBin-flow has lower recall than MOB-recon and at times, plasmidSPAdes and gplas. In this section, we take a closer look at this issue, by introducing additional plasmid-specific statistics related to recall. The statistics we present were obtained on the 66 samples of our test data set.

Let T_v be a true plasmid and P_u a predicted plasmid: we denote by overlap(P_u, T_v) the contribution of contigs in the intersection of these two sets, either in terms of number of contigs or in terms of their cumulative length. Given a true plasmid T_v, the predicted plasmid bin $P_g(v)$ that maximizes the overlap with T_v, is called its match. All predicted plasmid bins that have at least one contig common with plasmid T_v form its neighbourhood N_v.

The recall for a true plasmid is defined as

$$\frac{overlap\ (P_g(v), T_v)}{size\ (T_v)}.$$

where *size* is the number of contigs for unweighted statistics and their summed lengths for weighted statistics.

Low recall indicates that contigs that belong to a true plasmid T_v are missing from its match $P_g(v)$. This can happen due to two reasons.

- *Misindentification*: PlasBin-flow did not identify the relevant contigs as plasmid contigs (i.e. include them in a plasmid bin);
- *Misbinned contigs*: PlasBin-flow placed the contigs in bins other than $P_g(v)$.

  Misbinning of contigs can occur as one of two cases:

  – *Splitting*: The contig set of T_v is split into multiple bins without contigs belonging to other true plasmids.

  – *Scrambling*: The contig sets of multiple true plasmids have been mixed or scrambled into several plasmid bins.

  We believe that it is relevant distinguishing between these different causes for recall errors, as splitting and misidentification are errors that are likely to be easier to correct (e.g. through post-processing) than scrambling errors.

In order to determine the extent of misidentification, splitting and scrambling, we use the following definitions and formulae, for a given true plasmid T_v:

- *Overall Recall* ($R_{overall}$): Proportion of contigs from T_v, identified in at least one predicted bin. Essentially, this gives us the recall if we were identifying plasmidic contigs rather than binning.

$$\frac{size\ (\cup_{u \in N_v}(P_u \cap T_v))}{size\ (T_v)}$$

- *Unidentified* (U): Proportion of contigs from T_v, not identified in any predicted bin. Thus,

$$U = 1 - R_{overall}$$

  .

- *Misbinned* (M): Proportion of contigs from T_v, identified but not present in $P_g(v)$. Thus,

$$M = R_{overall} - R_{match}.$$

  Here, R_{match} is the recall reported by considering only the matched plasmid bin.

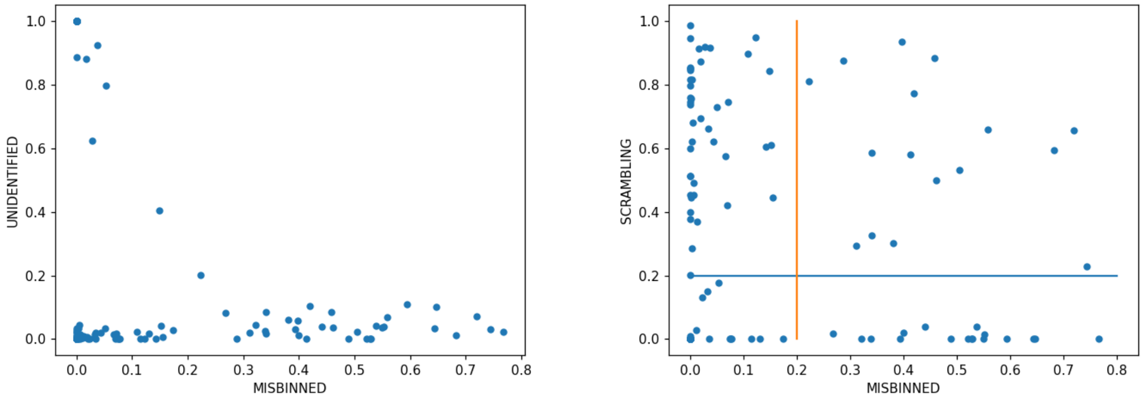

Figure 9: (Left) Misbinning vs. misindentification. (Right) Misbinning vs. scrambling

- *Scrambling* (S): Contigs not from T_v but present in some bin in N_v. The scrambling statistic is computed as the ratio of the total length of these extra contigs to the cumulative length of all contigs present in at least one of the bins in N_v.

$$\frac{size\ (\cup_{u \in N_v}(P_u - T_v))}{size\ (\cup_{u \in N_v} P_u)}$$

Figure 9 indicates that the (weighted) proportion of contigs that have been misindentified by PlasBin-flow is typically low barring a few exceptions. The recall is mostly affected by misbinning. For quite a few true plasmids, the loss of recall due to misbinning is more than 0.5. This means more than half of the contigs in the true plasmid were scattered across 2 or more bins, not including the matched bin; this indicates a higher level of splitting and/or scrambling.

The statistic for misbinning is affected by both splitting and scrambling. When the level of scrambling is low, misbinning seems to be due to splitting. Figure 9 indicates that there are rare cases when the scrambling factor being high contributes to the misbinning. In most cases with high scrambling, the misbinning is low. In such cases, the impact on recall is relatively low. There are cases (top right section) where both misbinning and scrambling is high: 15 out of 147 true plasmids face this issue. However, there is a significant number of true plasmids with high misbinning and low scrambling (bottom right section). Splitting is the main cause of poor recall in these cases.

References

[1] Garth McCormick. Computability of global solutions to factorable nonconvex programs: Part I — convex underestimating problems. *Mathematical programming*, 10(1):147–175, 1976.
